# Beneficial Effect of Glucose Control on Atherosclerosis Progression in Diabetic ApoE^−/−^ Mice: Shown by Rage Directed Imaging

**DOI:** 10.1155/2014/695391

**Published:** 2014-04-14

**Authors:** Yared Tekabe, Maria Kollaros, Qing Li, Geping Zhang, Chong Li, Ann Marie Schmidt, Lynne L. Johnson

**Affiliations:** ^1^Department of Medicine, Columbia University Medical Center, 650 West 168 Street, New York, NY 10032, USA; ^2^Department of Medicine, New York University Medical Center, New York, NY 10016, USA

## Abstract

*Objective.* Receptor for advanced glycated endproducts (RAGE) plays an important role in atherogenesis in diabetes. We imaged RAGE to investigate the effect of glucose control to suppress RAGE and reduce atherosclerosis in apolipoprotein E null (apoE^−/−^) diabetic mice. *Methods and Results.* Thirty-three apoE^−/−^ mice received streptozotocin and 6 weeks later 15 began treatment with insulin implants. Blood glucose measurements during study averaged: 140 ± 23 mg/dL (treated) and 354 ± 14 mg/dL (untreated). After 15 wk 30 mice were injected with ^99m^Tc-anti-RAGE F(ab′)_2_, 3 with ^99m^Tc-nonimmune IgG F(ab′)_2_, and all with CT contrast agent and underwent SPECT/CT imaging. At necropsy, the proximal aorta was weighed, counted, and sectioned and the % injected dose per gram (%ID/g) was calculated. From the merged SPECT/CT scans, tracer uptake localized to arteries was lower in the treated mice: 3.15 ± 1.82 × 10^−3^ versus 8.69 ± 4.58 × 10^−3^%ID (*P* = 0.001). Percent cross-sectional lesion area was smaller in the treated (14.3 ± 7.8% versus 29.5 ± 10.9%) (*P* = 0.03). RAGE uptake on scans (%ID) correlated with quantitative RAGE staining in the atheroma and with %ID/g (*R* = 0.6887; *P* = 0.01). Lesion size as percent cross-sectional area was smaller in the treated (14.3 ± 7.8% versus 29.5 ± 10.9%) (*P* = 0.03). RAGE uptake on scans (%ID) correlated with quantitative RAGE staining in the atheroma and with %ID/g (*R* = 0.6887; *P* = 0.01). *Conclusions.* These results support the importance of suppressing RAGE to reduce atherosclerotic complications of diabetes and value of molecular imaging to assess treatment effect.

## 1. Introduction

Type I diabetes is associated with increased risk of atherosclerotic macrovascular disease [1, 2]. While nonglycemic risk factors associated with the metabolic syndrome contribute to risk for cardiovascular (CV) events in type II diabetes, hyperglycemia has an independent effect to increase risk [2–4]. The nonenzymatic reaction between glucose and proteins known as glycation or glycosylation produces increased levels of glycoxidation products or advanced glycation endproducts (AGEs) in vascular endothelial cells and extracellular space. AGEs as well as other inflammatory ligands interact with a multiligand cell receptor (receptor for advanced glycation endproducts—RAGE) and initiate a positive feedback loop whereby more binding leads to further RAGE upregulation. RAGE and its ligands play a critical role in vascular inflammation, endothelial dysfunction, and atherosclerotic plaque development [5–7].

In an autopsy study of coronary arteries from sudden cardiac death subjects, greater RAGE expression was found in coronary atheroma from diabetic compared to nondiabetic subjects [8]. Analysis of carotid plaques from patients with and without diabetes showed RAGE expression associated with inflammatory cells and matrix metalloproteinase (MMP) expression, markers of vulnerability, and correlated linearly with levels of glycated hemoglobin HgA1c [9]. Glycemic control has been shown to reduce the risk of CV events in insulin dependent type I diabetes [1, 10]. While results from multicenter prospective trials have shown either no effect or a deleterious effect of glucose control on CV events in type II diabetes, when patients with earlier stage disease and no history of overt events were analyzed separately a beneficial effect of glucose control was found [11].

In murine models of atherosclerosis with either type I or type II diabetes, atherosclerotic lesion development is accelerated compared to atherosclerosis alone [12–14]. Treating with soluble RAGE (s-RAGE) that binds ligands and thereby decreases RAGE expression reversed the effect of diabetes to accelerate lesion growth in apolipoprotein E null (apoE^−/−^) diabetic mice [15, 16]. Soluble RAGE is not available as a treatment option in patients. We undertook this study to investigate in live animals using molecular imaging the effects of glucose control with insulin on RAGE expression and atherosclerotic lesion size and progression.

## 2. Materials and Methods

### 2.1. Animals

All animal experiments were performed with the approval of the Institutional Animal Care and Use Committee of Columbia University. ApoE^−/−^ mice with the genetic background of C57BL/6 mice were purchased from The Jackson Laboratories (Bar Harbor, Maine). At 6 weeks of age, mice (*n* = 30) were made diabetic via 5 daily intraperitoneal injections of streptozotocin (STZ, Sigma), 50 mg/kg in citrate buffer (0.05 mol/L, pH 4.5) as described elsewhere [17]. Blood glucose levels were monitored weekly via tail vein sampling using blood glucose monitor (FreeStyle Lite, Abbott).

After 6 weeks of diabetes, fifteen mice received implants (LinBits, LinShin Canada Inc., Toronto, Canada) that release insulin at a rate of ~0.1 U/day/implant for >30 days. Briefly, mice were anesthetized with isoflurane (4% to induce, 1% to maintain) and the insulin implant was briefly immersed in 2% betadine and implanted subcutaneously using a 12-gauge trocar. The number of initial implants was determined based on weight: 2 LinBit implants for the first 20–24 g in body-weight and a third weight ≥25 g. The average weight for the mice was 28 g and mice received 2-3 implants. Blood glucose levels were monitored subsequently at one-week intervals. Food was removed in the morning before the blood sample was obtained. If the glucose was <144 mg/dL food was restored immediately. If the glucose level was >288 mg/dL, additional implants were inserted based on blood glucose level. Water intake was monitored and the bottle changed 2-3 times a week. Mice were monitored for signs of dehydration, weight loss, and loss of grooming which none of the mice in this study developed. Over the course of the 15 weeks mice received an average of 7 implants (range 6–11).

Three additional untreated mice were used for control nonimmune antibody experiment. In addition, blood pool clearance for ^99m^Tc-anti-RAGE  F(ab′)_2_ was measured in apoE^−/−^ mice at 34 weeks with larger lesions and at 20 weeks with small lesions.

### 2.2. Radiotracer Preparation

Monoclonal anti-RAGE antibody was developed as previously described [17, 18]. The antibody fragments were labeled with ^99m^Tc using DTPA [19]. An aliquot of anti-RAGE F(ab′)_2_ (1 mg) reacted with 5-fold molar excess of bicyclic anhydride of diethylenetriaminepentaacetic acid (DTPA) in 0.5 mL of dimethyl sulfoxide (DMSO) for 30 min at room temperature while stirring. The reaction mixture was dialyzed against excess (4 L) 0.1 mol/L NaHCO_3_ in 0.1 mol/L NaCl, pH 7.6 at 4°C overnight. An approximate 50–100 *μ*g aliquot of DTPA modified anti-RAGE F(ab′)_2_ reacted with 30 mCi (1100 MBq) of ^99m^TcO_4_^−^ in 50 *μ*g of SnCl_2_ in 100 *μ*L of 0.1 N HCl that was flushed with N_2_ for 20 min. After 30 min of incubation, the ^99m^Tc-anti-RAGE F(ab′)_2_ was separated from free ^99m^Tc by Sephadex-G25 (10 mL) column (Pharmacia) equilibrated with PBS. Fractions (1.0 mL) were collected, and those fractions containing ^99m^Tc-anti-RAGE F(ab′)_2_ in the void volume were pooled. The mean specific activity was 48.7 ± 9.3 *μ*Ci/*μ*g (1.8 ± 0.34 MBq/*μ*g), and the mean radiochemical purity was 95 ± 1.6% by instant thin-layer chromatography. Nonimmune Mu IgG F(ab′)_2_ (Alpha Diagnostics) was similarly prepared.

### 2.3. Tracer Injection and Imaging

Each mouse was anesthetized with isofluorane (4% to induce, 1% to maintain) and injected with ^99m^Tc-anti-RAGE F(ab′)_2_ (15.14 ± 1.23 MBq) via femoral vein catheter and 4-5 hours later (allowing for radiotracer blood pool clearance) were injected with 0.1–0.15 mL of CT contrast agent (eXIA 160) (Binitio Biomedicals, Ottawa ON). For control antibody experiment, ^99m^Tc-mouse nonimmune IgG F(ab′)_2_ (18.64 ± 0.4 MBq) was injected in 3 untreated mice.

CT images were acquired with an integrated CT scanner using an X-ray tube at 45 kVp and an exposure time of 1000 ms per view. Following CT acquisition, helical SPECT scans were acquired using dual-headed detectors each outfitted with collimators with nine pinholes. Each pinhole has a diameter of 1.4 mm with each collimator providing a transaxial field-of-view (FOV) of 70 mm and an axial FOV up to 35 mm. SPECT data were acquired with the following parameters: step and shoot rotation, 30° step in 360° rotation using 24 projections, 60 s per projection, 256 × 256 frame size with 1.0 mm pixels, and 140 keV with 10% energy window. The projection data were reconstructed by ordered subset expectation-maximization (OSEM) algorithm with subset and iteration number set to 16 and 8, respectively, and a voxel size of 300 *μ*m and SPECT and CT datasets fused. Following imaging, mice were euthanized by an intraperitoneal injection of pentobarbital (100 mg/kg).

### 2.4. Image Analyses

**Figure 1:**
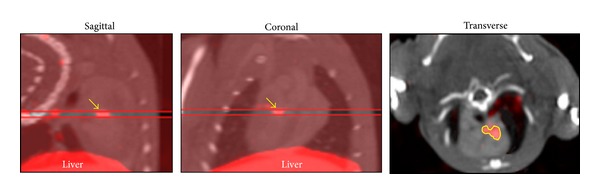
Method for ROI section. Left and center images show slice range chosen to comprise cephalocaudal limits of focal area of tracer uptake in proximal aorta (arrows) and right image shows region (yellow line) denoting area boundaries of the 5-voxel-thick slice from which activity in mCi is determined using software algorithm (see text).

The scans were reconstructed and processed using InVivoScope software (Invicro, Boston, MA). The cephalocaudal limits of tracer uptake in the ascending aorta and arch and brachiocephalic vessels were identified from coronal and/or sagittal images using contrast in the vessels on the CTA for anatomical localization. Regions of interest (ROIs) were then drawn around focal uptake in the transverse image and counts in these volumetric ROIs converted to mCi using a calibration standard and conversion algorithm (Figure 1). Quantification of tracer uptake in mCi in the reconstructed SPECT images was performed in Interview XP software (Mediso) using region-of-interest drawings in the transaxial plane. The system is calibrated for absolute quantification using specially designed rat- and mouse-shaped phantoms filled with known levels of ^99m^Tc imaged with the same protocol used for the animal studies. This quantification technique has been shown to be better than 4% accurate when performed with the same methodology (HiSPECT and Mediso software) on a comparable imaging system for imaging targets including vascular lesions [17]. The quantitative uptake in mega-Becquerel's was divided by total injected dose (decay corrected) to obtain %ID. The summed activity from all the volumetric ROIs for the ascending aorta and arch was correlated with ex vivo gamma counting of the same tissue. To compare total vascular uptake of tracer on scans between groups, all tracer uptakes including the brachiocephalic vessels were summed for each animal.

To confirm that blood glucose levels did not affect blood pool activity of the anti-RAGE probe at the time of imaging and contribute to differences in lesion uptake, using the CTA scans to identify the LV cavity, uniform sized ROIs were drawn in the center of the LV cavity on transverse slices. Activity in these samples of LV cavity blood pool was measured as %ID applying the calibration hardware/software as described above.

### 2.5. Gamma Well Counting

The proximal aorta (ascending aorta and arch) was dissected and weighed and the radioactivity was determined in a gamma well counter (Wallac Wizard 1470, PerkinElmer, Waltham, MA) and expressed as the percentage of injected dose per gram (%ID/g) of tissue. The radiotracer activity in the samples was corrected for background, decay time, and tissue weight.

### 2.6. Histopathology and Quantitative Morphometry

The proximal aorta was fixed for 24 h in formalin (10%) and was paraffin embedded. Tissue blocks were sectioned (5 *μ*m-thick) and stained with hematoxylin and eosin (H&E) for morphology. For immunohistochemical analyses, serial sections were deparaffinized in xylene and treated with 0.3% hydrogen peroxide for 20 min, followed by incubation in protein-free block (Dako, Carpinteria, CA) for 10 min to inhibit the nonspecific binding of primary antibody. Staining for RAGE was performed using monoclonal anti-RAGE antibody (50 *μ*g/mL). Macrophages were identified using marker Mac-3 (1 : 20, BD Pharmingen). Secondary staining was performed with HRP-conjugated respective secondary antibody, followed by diaminobenzidine (DAB substrate kit for peroxidase, Vector Laboratories) and counterstaining with Gill's hematoxylin solution.

Morphometric and immunohistochemical analyses of the arterial segments were performed using a Nikon microscope and Image-Pro Plus software (Media Cybernetics Inc., Silver Spring, MD). The lesion in the proximal aorta was measured from the H&E stained sections as percent lesion area per total area of the aorta. Staining for RAGE and macrophages was quantified as area with positive staining in the lesion over total cross-sectional area of the aorta.

### 2.7. Statistical Analysis

All data are presented as mean ± standard deviation. Statistical comparison between groups was made by use of either paired or unpaired Student's *t*-test. Correlation was assessed using the Pearson product-moment correlation coefficient. Differences between groups were considered significant at a value of *P* < 0.05.

## 3. Results

### 3.1. Glycemic Control

**Figure 2:**
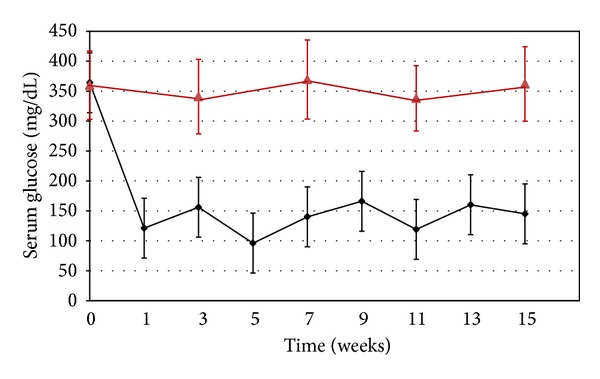
Chart showing mean ± SD for serum glucose levels for the apoE^−/−^ diabetic mice treated with insulin from time 0 (after 6 weeks of diabetes) through week 16 (black line) and untreated control mice (red line).

The mean serum glucose levels for the apoE^−/−^ mice with 6 weeks of diabetes and prior to insulin treatment were 364 ± 66 mg/dL for the mice that received the insulin implants and 360 ± 57 mg/dL for the mice that were followed as hyperglycemic controls. The glucose values for the treated and untreated mice are shown in Figure 2. The initial weight of the mice that received the implants averaged 27 ± 3 g. The average of weekly glucose levels over the 15-week treatment period for the insulin treated mice was 140 ± 23 mg/dL and for the untreated hyperglycemic controls was 354 ± 14 mg/dL.

### 3.2. Extent of Atherosclerosis at Necropsy

**Figure 3:**
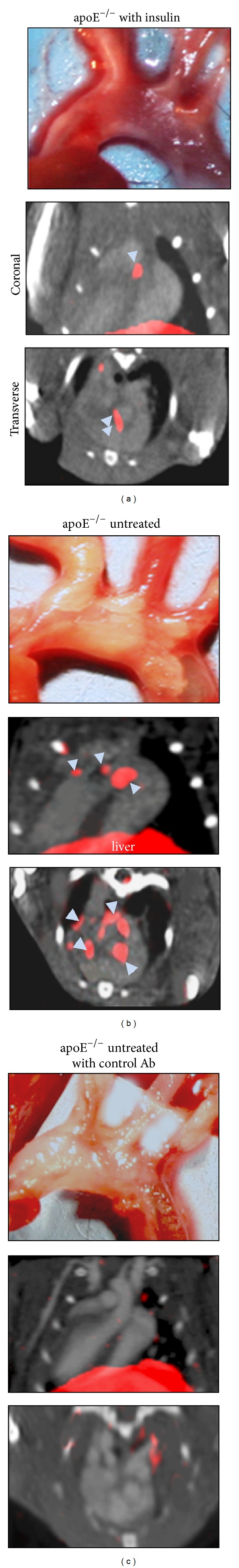
Atherosclerotic lesions and SPECT/CT scans. Each panel shows in situ dissection on top and in middle and lower panels coronal and transverse slices from SPECT/CT scans with IV contrast. Panel (a) is an example of a treated mouse and panel (b) is an example of an untreated mouse injected with ^99m^Tc-anti-RAGE F(ab′)_2_ and panel (c) is an example of an untreated mouse injected with control antibody. There is less extensive atheroma in the treated mouse compared to the two untreated mice. The CT contrast delineates the ventricular cavities and arterial vessels. Regions of uptake of the ^99m^Tc-anti-RAGE F(ab′)_2_ (blue arrows) are seen localized to the proximal aorta in the treated mouse and are more extensive in the ascending aorta and arch in the untreated mouse. No uptake is seen in the mouse injected with control antibody despite extensive atherosclerotic disease.

At necropsy there was extensive atherosclerosis involving the ascending aorta and arch and extending into the brachiocephalic and carotid vessels in the untreated mice. In the treated mice, the lesions were smaller and confined to the ascending aorta and arch (Figure 3).

### 3.3. Analysis of Radiolabeled Anti-RAGE Probe

**Figure 4:**
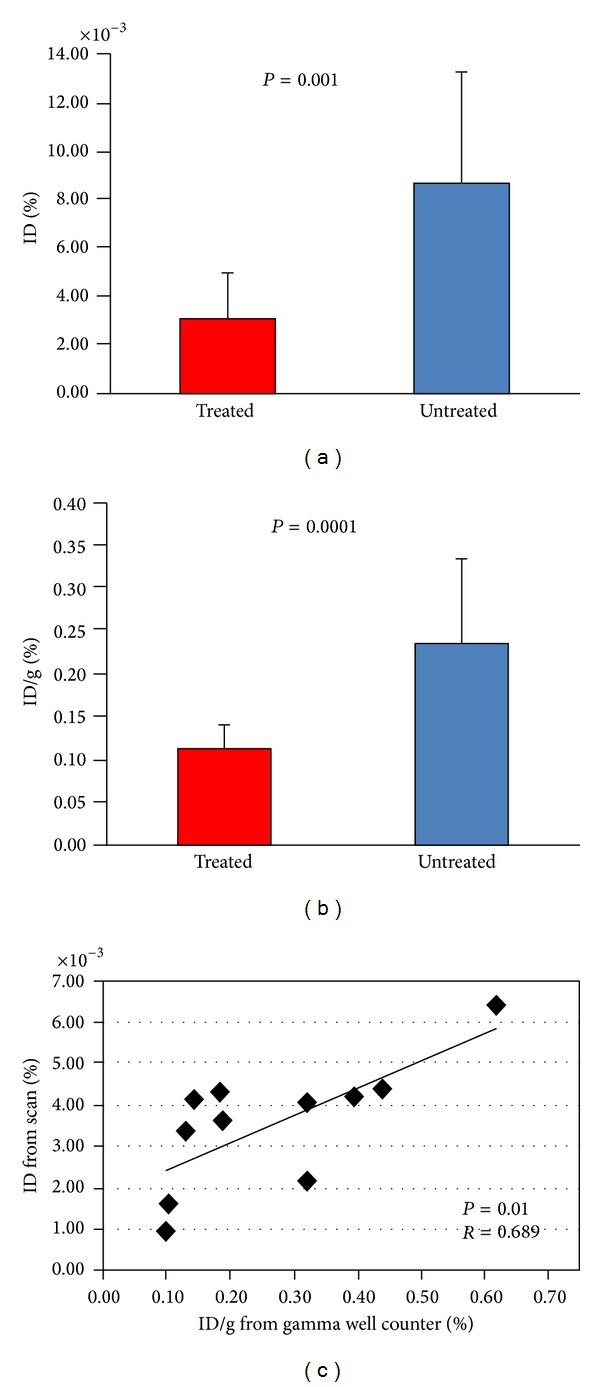
In vivo and ex vivo activity. (a) Scan measurements of %ID as mean ± SD in insulin treated apoE null diabetic mice (red bar) and untreated diabetic apoE null mice (blue bar). (b) Ex vivo well counting values for same groups. (c) Correlation for %ID from scans versus %ID/g from tissue counting.

All insulin treated mice showed lower tracer uptake in the thorax and neck corresponding to the location of the proximal aorta, arch, and brachiocephalic branches on the coregistered CT scan compared with untreated mice. An example from each experiment is shown in Figure 3. Using the method described above to measure tracer uptake as %ID, the average was significantly lower in the glycemic controlled mice (3.15 ± 1.82 × 10^−3^) compared to the uncontrolled mice (8.69 ± 4.58 × 10^−3^) (*P* = 0.001) (Figure 4). Uptake measured from the ascending aorta and arch alone was 6.49 ± 3.87 × 10^−3^ in the untreated group and also significantly higher than for the same segments for the treated group (*P* = 0.02). None of the treated mice showed uptake in the brachiocephalic branch vessels, while all but 2 of the untreated mice did. Uptake in the brachiocephalic and carotids in the untreated mice was 2.71 ± 2.46 × 10^−3^. Minimal to no focal uptake of tracer was seen in these vascular territories on scans from untreated diabetic mice injected with control antibody (Figure 3). Average value for %ID for these mice was 0.59 × 10^−3^.

Analysis of LV cavity ROIs as representing samples of blood pool activity showed that blood glucose levels did not contribute to the observed differences in lesion uptake. Values for the treated group were 0.28 ± 0.17 × 10^−4^ and for the untreated group 0.39 ± 0.36 × 10^−4^ (*P* = 0.47).

### 3.4. Gamma Well Counting

The lower tracer uptake in the proximal aorta and arch of the treated mice was confirmed by ex vivo gamma well counting. Uptake of the radiotracer in the treated group was 0.11 ± 0.03%ID/g which is significantly lower than uptake in the untreated group (0.24 ± 0.10%ID/g) (*P* < 0.0001) (Figure 4). Radiotracer uptake as %ID/g for the control antibody was 0.07 ± 0.01%.

Regression analysis of individual values for tracer uptake in the ascending aorta and arch from scans (%ID) and ex vivo gamma counts (%ID/g) showed significant correlation (*r* = 0.69; *P* = 0.01) (Figure 4).

### 3.5. Lesion Size

The mean cross-sectional area of the proximal aortic lesions, expressed as percent lesion area over total aortic area, in the insulin treated group (14.2 ± 7.8%) was significantly smaller than the untreated mice (27.7 ± 8.3%; *P* = 0.01).

### 3.6. Quantitative Immunohistology

**Figure 5:**
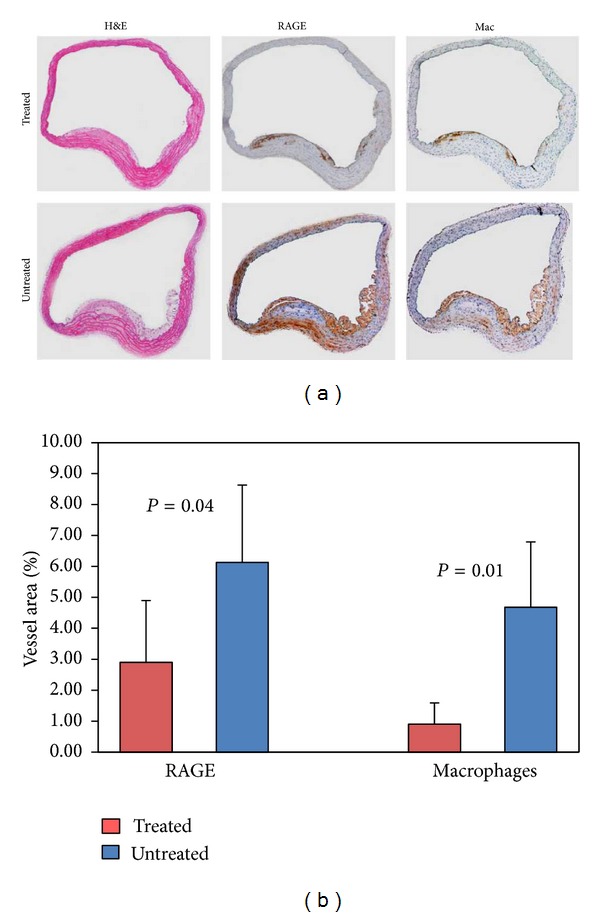
Cross-sections of aortic tissue stained for H&E (left), RAGE (middle), and macrophages (right) for treated mouse (top row) and untreated mouse (bottom row). The untreated mouse shows a larger lesion size and greater staining for both RAGE and macrophages. Graph depicts results of quantitative histomorphometry showing significantly lower area of cells staining + for both RAGE and macrophages in the treated group.

Histological sections through the proximal aorta showed less staining for RAGE expressed as % positive staining cell area divided by total vessel area in the treated group (2.9 ± 2.0%) compared with untreated group (6.13 ± 2.5%; *P* = 0.04) (Figure 5). Serial sections stained for macrophages also showed less staining in the treated group (0.80 ± 0.69) compared with untreated group (4.68 ± 2.11) (*P* = 0.01).

## 4. Discussion

The results of this study show the beneficial effect of reducing RAGE expression by glycemic control on atherogenesis in diabetic apoE^−/−^ mice. This reduction in RAGE expression in the insulin treated mice as documented by lower uptake of ^99m^Tc-anti-RAGE F(ab′)_2_ in the proximal aorta was associated with less extensive disease at necropsy and fewer lesion macrophages. None of the treated mice showed tracer uptake in the brachiocephalic vessels, while all but 2 of the untreated mice showed uptake in these vessels. These data support the value of in vivo imaging to document the effects of an intervention on target expression and disease progression.

Overexpression of RAGE in vascular endothelial cells and monocytes plays a critical role in development of atherosclerosis in nondiabetic apoE^−/−^ mice [9] and with the addition of diabetes atherosclerosis development is accelerated [12–14]. Similar effect of RAGE expression on atherosclerosis was shown in apoE^−/−^ db/db mice, a murine model of type II diabetes [14]. Blocking RAGE with soluble RAGE (s-RAGE) reduced the rate of atherogenesis to the nondiabetic pattern [15, 16]. In these experimental studies RAGE expression was measured using quantitative immunohistology or Western blots on tissue samples. A radiolabeled antibody probe allows quantification of RAGE expression in live animals. We have shown that uptake of the antibody as %ID correlates with both quantitative immunohistological staining for RAGE in vascular tissue and with atheroma size in apoE^−/−^ mice and that RAGE colocalizes with both endothelial cells and monocytes in the atherosclerotic plaque [17, 18]. In this current study we have shown that this imaging approach provides quantitative data on effects of treatment to reduce RAGE expression.

Epidemiological studies linking hyperglycemia to macrovascular atherosclerosis in diabetes have implicated glycated endproducts through inflammatory pathways and suggest that lowering glucose should reduce atherogenesis in diabetic patients. The Diabetes Control and Complications Trial (DCCT) randomly assigned 1441 patients with type I diabetes to “intensive” or “conventional” insulin therapy. They first reported that intensive glucose control delays the onset and slows the progression of microvascular complications of retinopathy, nephropathy, and neuropathy [1]. Further study of the same cohort for CV events reported in 2005 showed that intensive therapy defined by strict fasting and low postprandial glucose levels conferred a long-term reduction in risk from CV events [10]. In our animal model, apoE^−/−^ mice were diabetic for 6 weeks before beginning insulin treatment. The extent and severity of atherosclerosis in 12-week apoE^−/−^ mice with 6 weeks of diabetes are minimal and therefore beginning insulin at this time point corresponds to early treatment. While directly relating this model to human disease is difficult, the closest fit would be to establish and maintain glucose control in young patients with type I diabetes.

We found RAGE staining on aortic tissue colocalized with macrophage staining and quantitatively fewer macrophages were found in the atheroma of insulin treated compared to the hyperglycemic controls. Immunohistological analysis of human atherosclerotic plaque showed that RAGE expression correlated with poor glucose control and with plaque inflammation [9]. While we cannot conclude that RAGE was the only inflammatory pathway contributing to atherogenesis affected by glucose lowering, the established link between hyperglycemia and nonenzymatic glycosylation of proteins to produce the AGE ligands that bind to RAGE and stimulate further RAGE expression suggests that lowering blood glucose lowers circulating AGEs and reduces ligand binding and receptor mass which in turn reduces RAGE activated downstream inflammatory pathways. This effect we were able to detect using in vivo molecular imaging was associated with reduction in atherogenesis when compared with hyperglycemic controls.

*Limitations.* We did not perform autoradiography on explanted tissue in this study but we have previously shown using dual-labeled probe (fluorescent plus radionuclide) the colocalization of the anti-RAGE F(ab′)_2_ and fluorescent probe in the atheroma [17]. While the spatial resolution of nuclear imaging is less than CT or MRI, the small probe size of a nuclear tracer allows access to binding sites within the plaque, and “hot spots” on in vivo imaging can be localized and quantified. Using consistent techniques for designating regions of interest, activity which relates to target expression can be assessed in live animals allowing group comparisons at single time points and serial time points in the same animal.

## 5. Conclusion

These data further support the important role of RAGE expression in atherogenesis in diabetes and support the value of molecular imaging to demonstrate and quantify responses to therapies targeting RAGE expression in atherosclerosis.
